# Recurrence of allergic bronchopulmonary aspergillosis after adjunctive surgery for aspergilloma: a case report with long-term follow-up

**DOI:** 10.1186/s12890-018-0743-0

**Published:** 2018-12-04

**Authors:** Kohei Horiuchi, Takanori Asakura, Naoki Hasegawa, Fumitake Saito

**Affiliations:** 1 Department of Pulmonary Medicine, Eiju General Hospital, 2-23-16 Higashi Ueno, Taito-ku, Tokyo, 110-8645 Japan; 2 Division of Pulmonary Medicine, Department of Medicine, Keio University School of Medicine, Tokyo, Japan; 3 Department of Mycobacteriology, National Institute of Infectious Diseases, Tokyo, Japan; 4 Center for Infectious Diseases and Infection Control, Keio University School of Medicine, Tokyo, Japan

**Keywords:** Allergic bronchopulmonary aspergillosis, *Aspergillus*, Pulmonary *Aspergillus* overlap syndrome, Relapse

## Abstract

**Background:**

Coexistence of aspergilloma and allergic bronchopulmonary aspergillosis (ABPA) has rarely been reported. Although the treatment for ABPA includes administration of corticosteroids and antifungal agents, little is known about the treatment for coexisting aspergilloma and ABPA. Furthermore, the impact of surgical resection for aspergilloma on ABPA is not fully understood. Here, we present an interesting case of recurrent ABPA with long-term follow-up after surgical resection of aspergilloma.

**Case presentation:**

A 53-year-old man with a medical history of tuberculosis was referred to our hospital with cough and dyspnea. Imaging revealed multiple cavitary lesions in the right upper lobe of the lung, with a fungus ball and mucoid impaction. The eosinophil count, total serum immunoglobulin E (IgE), and *Aspergillus*-specific IgE levels were elevated. Specimens collected on bronchoscopy revealed fungal filaments compatible with *Aspergillus* species. Based on these findings, a diagnosis of ABPA with concomitant aspergilloma was made. Although treatment with corticosteroids and antifungal agents was administered, the patient’s respiratory symptoms persisted. Therefore, he underwent lobectomy of the right upper lobe, which resulted in a stable condition without the need for medication. Twenty-three months after discontinuation of medical treatment, his respiratory symptoms gradually worsened with a recurrence of elevated eosinophil count and total serum IgE. Imaging revealed recurrent bronchiectasis and cavities with mucoid impaction in the right lower lobe, suggesting relapse of aspergilloma and ABPA. Corticosteroids and antifungal agents were re-administered; aspergilloma improved slightly over a 5-year period, and ABPA remained well controlled with low-dose prednisolone (5 mg/day).

**Conclusions:**

We describe the long-term follow-up outcomes of a patient with concomitant ABPA and aspergilloma, who underwent surgical resection for aspergilloma. Physicians should carefully monitor patients with coexisting ABPA and aspergilloma, as the condition may relapse after remission, even despite surgical resection for aspergilloma. Additionally, surgical resection for aspergilloma could result in resolution of ABPA.

## Background

*Aspergillus* is a ubiquitous fungus isolated from both outdoor and indoor environments, including hospitals. Although *Aspergillus* spores are inhaled daily, only a minority of the population consequently develops pulmonary disease. Depending on the interaction between the fungal burden and host’s immune status or immune hyperactivity, pulmonary aspergillosis has a wide spectrum of disease presentations, including chronic pulmonary aspergillosis such as aspergilloma and chronic necrotizing aspergillosis, invasive pulmonary aspergillosis, and allergic bronchopulmonary aspergillosis (ABPA) [1]. Aspergilloma is the most common form of the infection, and mostly occurs secondary to pre-existing cavitary conditions, such as previous pulmonary tuberculosis, bronchiectasis, and bronchial cysts [2], while ABPA, a hypersensitivity reaction to *Aspergillus* spp. that have colonized the lung, occurs almost exclusively in patients with asthma, cystic fibrosis, or chronic obstructive pulmonary disease [3].

The coexistence of ABPA and aspergilloma has infrequently been reported. A previous case series reported that eight of 179 (4%) ABPA patients had aspergilloma, which was associated with severe disease and relapse [4]. In terms of the treatment for aspergilloma, symptomatic patients, especially those with hemoptysis, are candidates for surgical resection, while observation is recommended for asymptomatic patients who do not demonstrate progression [5]. For ABPA, corticosteroids and anti-fungal agents are used to control inflammation and reduce fungal burden, respectively [5, 6]. However, little is known about the treatment for coexisting aspergilloma and ABPA [1]. Furthermore, the impact of surgical resection for aspergilloma on ABPA is not fully understood. Here, we present an interesting case of recurrent ABPA with long-term follow-up after surgical resection for aspergilloma.

## Case presentation

A 53-year-old man was referred to our hospital with purulent cough and progressive dyspnea of a few months’ duration. He had a history of tuberculosis at 31 years of age and had no other pulmonary diseases. He had never smoked cigarettes.

**Fig. 1:**
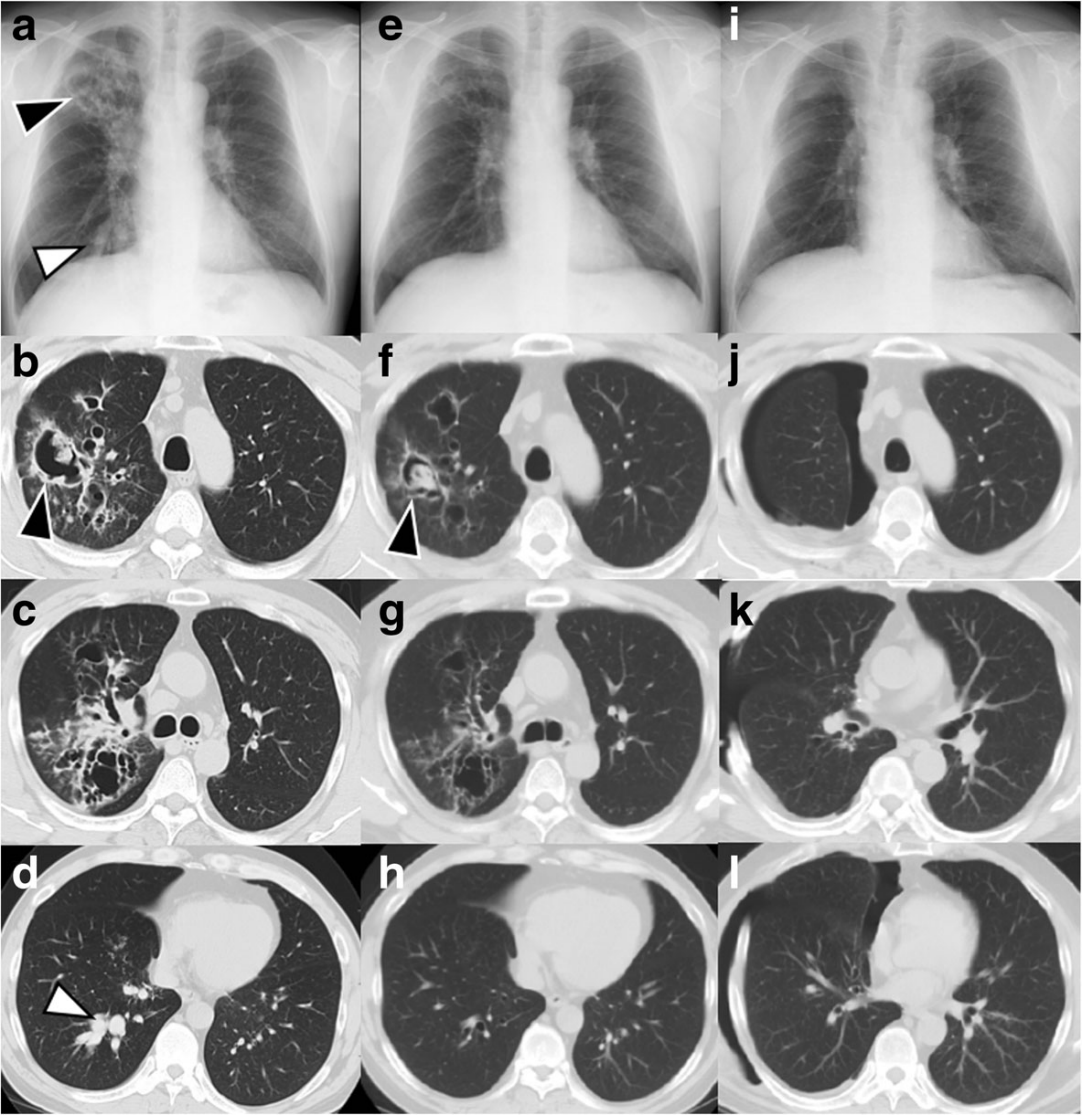
(**a**-**d**) Chest imaging on admission showed cavitary lesions in the right upper lobe (black arrowhead) and mucoid impaction in the right lower lobe bronchi (white arrowhead). (**e**-**h**) Improvement was seen on chest imaging after administration of prednisolone and antifungal therapy; multiple cavities with fungus in the right upper lobe persisted (black arrowhead). (**i**-**l**) All the lesions improved after right upper lobe resection

Upon physical examination, chest auscultation detected coarse crackles from the right lung and slight wheezes, bilaterally. Chest radiography showed cavitary lesions in the right upper lung field and consolidation in the right lower lung field (Fig. 1a). Chest computed tomography (CT) revealed bronchiectasis and cavitary lesions with a fungus ball in the right upper lobe and mucoid impaction in the bronchi of the right lower lobe (Fig. 1b–d).

Laboratory examination revealed a total leukocyte count of 14,000 cells/μL (reference range 3500–8500 cells/μL) with 45.1% eosinophils (reference range 1–6%), elevated serum total IgE levels of 19,100 IU/ml (reference range < 173 IU/ml), elevated *Aspergillus*-specific IgE of 46.3 kUA/L (reference range < 0.35 kUA/L) by fluorescence-enzyme immunoassay, as determined at a commercial laboratory (SRL Inc., Tokyo, Japan).

Pathological examination of transbronchial lung biopsy specimens from the right B3 revealed fungal filaments compatible with *Aspergillus* species. Examination of bronchoalveolar lavage fluid (BALF) showed 3056 cells/μL with 70.5% eosinophils, 17.5% neutrophils, 10.5% macrophages, and 1.5% lymphocytes. Culture of sputum and BALF did not grow any fungus. Head and neck examination by fiberscope and magnetic resonance imaging revealed no evidence of sinusitis. Thus, ABPA with concomitant aspergilloma was diagnosed based on the International Society for Human and Animal Mycology criteria [7].

One month after referral, prednisolone (0.5 mg/kg/day) and itraconazole (ITC, 200 mg/day) were administered for ABPA. ITC was switched to voriconazole (VRC, 400 mg/day) 1 month later, as the patient’s symptoms and radiographic findings showed no improvement. Although the imaging findings revealed improvement of the cavitary lesions and mucoid impaction after 1 month (Fig. 1e–h), he still had cough and productive sputum. At the time, VRC was decreased to 200 mg/day due to liver dysfunction. To control the disease further, lobectomy of the right upper lobe was performed without any complication, 4 months after initiation of treatment. Pathological examination of the resected lobe revealed fungal filaments compatible with *Aspergillus* species without evidence of malignancy. The surgery resulted in gradual improvement of the patient’s symptoms, imaging findings (Fig. 1i–l), and serum total IgE levels. Due to his stable course, prednisolone and VRC were discontinued 5 and 7 months after surgery, respectively.

**Fig. 2:**
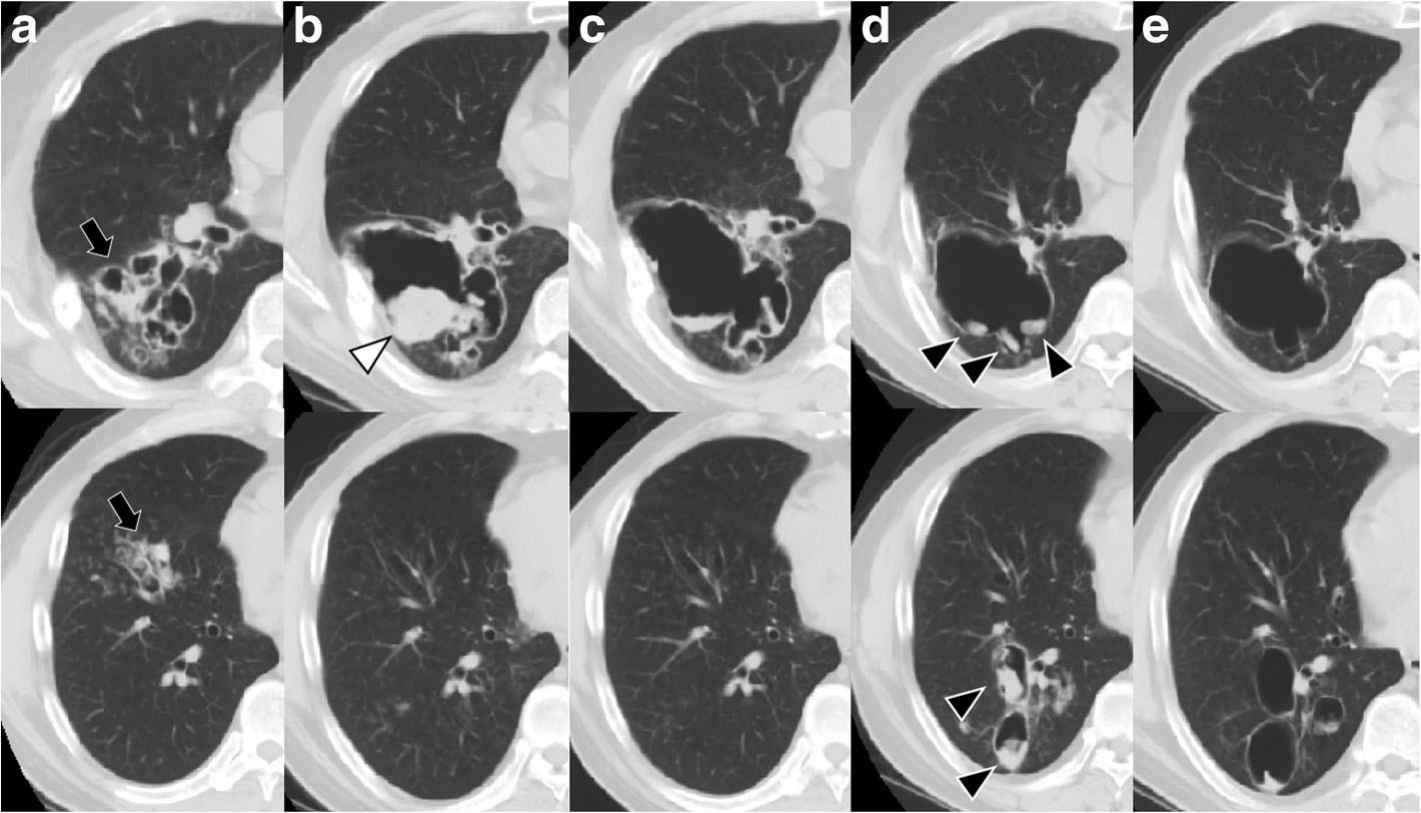
Chest computed tomography (CT) at the time of relapse (**a**), 2 months (**b**), 3 months (**c**), 22 months (**d**), and 6 years after the relapse (**e**). (**a**) Recurrence of bronchiectasis and cavities with mucoid impaction (black arrow) in the right lower lobe. (**b**) A cavitary lesion with a fungus ball (white arrowhead) was observed in the right lower lobe. (**c**) Improvement seen on CT after additional treatment with voriconazole. (**d**) CT scan showing advancement of fungus balls inside the large cavity in the right lower lobe (black arrowhead). (**e**) No fungus balls seen on CT 6 years after the relapse

Twenty-three months after discontinuation of the medical treatment, the patient complained of gradually worsening cough and dyspnea. Additionally, the patient’s eosinophil count and serum total IgE had been steadily increasing throughout the previous year. CT showed recurrent bronchiectasis and cavities with mucoid impaction in the right lower lobe (Fig. 2a). Bronchoscopy was performed and culture results of bronchial washings from the right lower lobe revealed no evidence of bacteria, mycobacteria, or fungus. Despite the lack of evidence of *Aspergillus* infection, prednisolone (0.5 mg/kg/day) was prescribed for relapsed ABPA, based on the elevated serum IgE and pathological CT findings. Two months after treatment was initiated, the patient remained symptomatic, and CT showed a cavitary lesion with fungus in the right lower lobe (Fig. 2b). Additional treatment with VRC (400 mg/day) for 3 months resulted in improvement of his symptoms and CT findings (Fig. 2c).

**Fig. 3:**
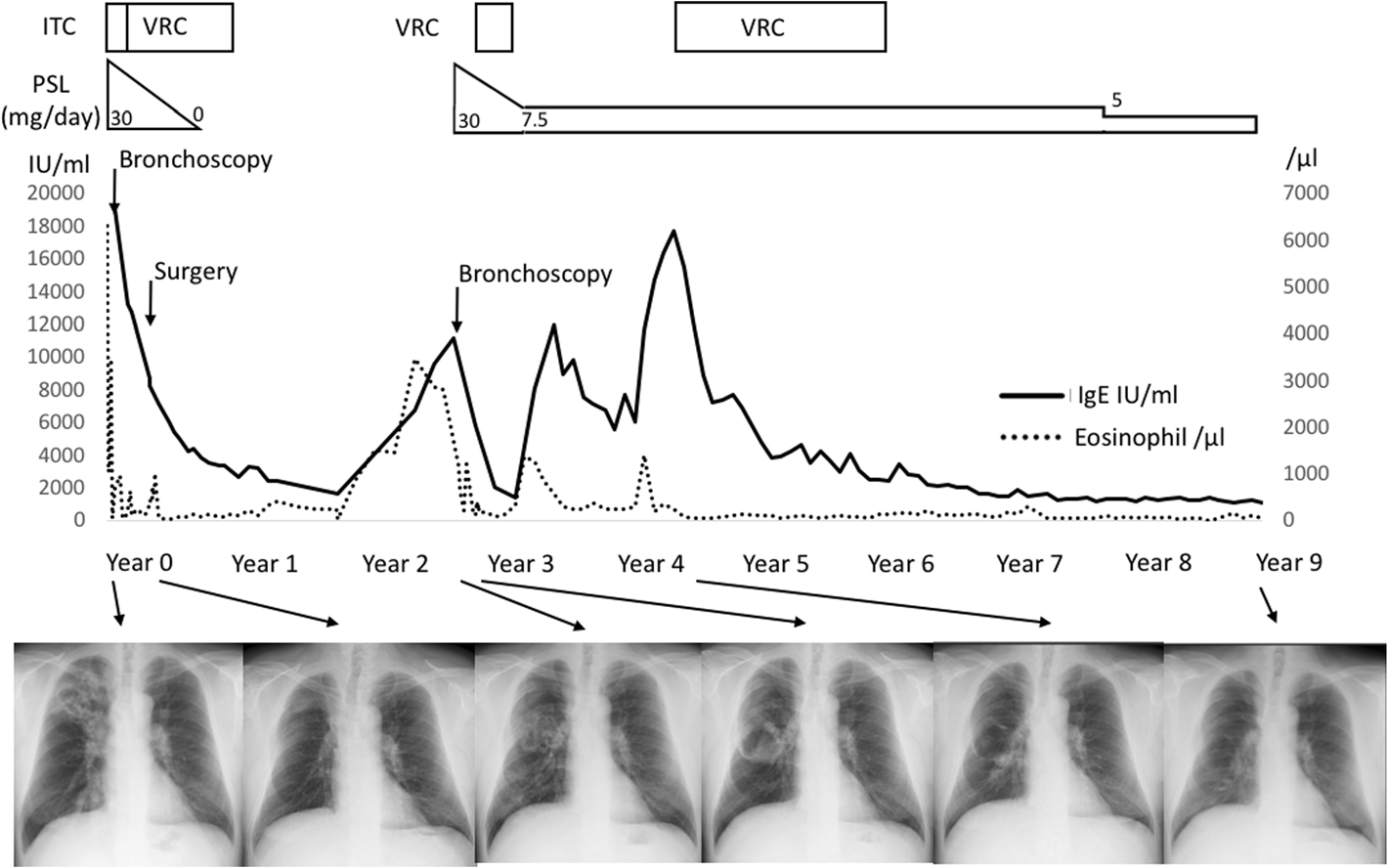
Clinical course of our case, including the changes in eosinophil counts (dotted line) and IgE levels (solid line). IgE; immunoglobulin E, ITC; itraconazole, VRC; voriconazole, PSL; prednisolone

Due to the markedly elevated serum total IgE, CT imaging was repeated 18 months after the re-treatment, although the patient’s symptoms had remained stable. CT revealed progression of the fungus balls inside the large cavity in the right lower lobe (Fig. 2d). The addition of VRC (400 mg/day) resulted in a decrease in the serum total IgE. VRC was discontinued after 20 months as the patient’s condition had become stable by that time. The aspergilloma improved slightly over a 5-year period (Fig. 2e), and ABPA remained well-controlled with low-dose prednisolone (5 mg/day). The summary of the treatment course is shown in Fig. 3.

## Discussion and conclusions

This case describes a recurrence of ABPA, with long-term follow-up after surgical resection for aspergilloma. The surgery resulted in ABPA remission without the use of corticosteroids and anti-fungal agents for about 2 years. However, the patient had recurrence of ABPA with aspergilloma in the residual lung. Eventually, re-administration of corticosteroids and adjunctive anti-fungal therapy achieved long-term remission of ABPA. This case highlights the need for careful monitoring of patients with coexistent ABPA and aspergilloma, as the condition may relapse after remission, even despite surgical resection for aspergilloma. The formation of aspergilloma may also be observed at the recurrence of ABPA. Additionally, surgical resection for aspergilloma could result in resolution of ABPA.

In our case, adjunctive surgery resulted in ABPA remission for 2 years. Previous studies have reported the outcome of surgical resection for aspergilloma in ABPA patients, due to pneumothorax [8] and hemoptysis [9, 10]. However, only one case involved a report of surgical resection for aspergilloma that contributed to disease control of ABPA; the patient was followed up without ABPA treatment for 6 months after receiving surgical resection for aspergilloma with antifungal therapy [11], whereas our case was followed up for a number of years.

Several pathogenic mechanisms have been suggested to be responsible for the overlap between ABPA and aspergilloma. On the one hand, in terms of aspergilloma development in cases of ABPA, in the early phase, bronchiectatic lesions affected by ABPA may enlarge to form cavities that become colonized with *Aspergillus* to create fungus balls [12, 13]. In the late phase, aspergilloma may form in patients with fibrosis and cavitation associated with long-standing or poorly treated ABPA. On the other hand, ABPA may develop due to a hypersensitivity reaction to an increased fungal burden in a patient with aspergilloma with pre-existing fibrocavitary disease, such as tuberculosis [14]. Thus, ABPA and aspergilloma have a synergistic relationship, which can result in a severe disease and recurrent relapse [4]. Furthermore, corticosteroids administered for the treatment of ABPA might accelerate the formation of aspergilloma [12] and lead to hemoptysis. Thus, adjunctive surgery for aspergilloma might be beneficial in ABPA patients as it reduces the fungal burden and allows the use of corticosteroid.

Although the patient had long-term remission after surgery, ABPA and aspergilloma relapsed concurrently, suggesting an overlap in the pathogenesis of these conditions in this patient. CT imaging (Fig. 2) showed severe bronchiectatic lesions with mucoid impaction, leading to the formation of cavities with aspergilloma. Previous studies in ABPA have reported a range of radiological features associated with recurrence or relapse, including extensive bronchiectasis, aspergilloma, and high-attenuation mucus [4, 15–17]. Notably, our patient relapsed even though the treatment, including surgery, resulted in initial improvement of all the pulmonary lesions caused by ABPA and aspergilloma (Fig. 1i–l). Therefore, maintenance therapy with corticosteroid or antifungal therapy might be needed to prevent relapse in high-risk patients, even after surgical resection of aspergilloma.

The treatment options for ABPA have included corticosteroid, anti-fungal therapy, and biologic agents, such as omalizumab and mepolizumab. In the acute phase, recent studies have suggested that medium-dose oral corticosteroid are as effective as and safer than high doses for the treatment of ABPA [18], and that ITC was also effective in a significant number of patients, with fewer side effects than prednisolone [19]. In our case, corticosteroids and ITC were simultaneously administered for concomitant aspergilloma, and we decided on surgical resection due to persistent symptoms, resulting in ABPA remission for 2 years. Although we could control ABPA by corticosteroid and anti-fungal therapy at the post-surgical relapse, other new therapies could be options for refractory cases. Recent studies have shown that omalizumab, a humanized anti-IgE monoclonal antibody, can be used safely for ABPA [20], and addition of mepolizumab, an anti-interleukin 5 monoclonal antibody, was also effective for refractory ABPA [21]. Furthermore, nebulized amphotericin could be beneficial for decreasing the frequency of exacerbations [22]. Since steroid therapy might deteriorate the formation of aspergilloma and further surgical resection may be limited, these treatments will be options in case of relapse.

The limitation of our case is that we were unable to detect the precise species of *Aspergillus* by culture of sputum, bronchial wash, or BALF. Additionally, molecular identification of fungal species was not available at our clinical site. Knowing the exact species and the resistance profile may have enabled us to provide better patient care in terms of the choice of antifungal agents. Although infrequent, *Aspergillus terreus* shows resistance to amphotericin B [23, 24]; is has been reported to cause ABPA and may form aspergilloma [25], which is reminiscent of our case. Another concern is that azole-resistant *Aspergillus fumigatus* is becoming a global health problem due to treatment failure and high mortality [26]. Regarding the treatment for azole-resistant *Aspergillus fumigatus*, most experts recommend a change from VRC monotherapy to liposomal amphotericin B or an azole–echinocandin combination [27]. These therapies are also considered in hospitals or wards where a 10% resistance threshold is exceeded [27].

In conclusion, we describe the long-term follow-up outcomes of a case with concomitant ABPA and aspergilloma, who underwent surgical resection for aspergilloma. This case emphasizes that physicians should carefully monitor patients with coexistent ABPA and aspergilloma, as the condition may relapse after remission, even after surgical resection for aspergilloma. Additionally, the case illustrates that surgical resection for aspergilloma could result in resolution of ABPA.

## Abbreviations

ABPA: Allergic bronchopulmonary aspergillosis
BALF: bronchoalveolar lavage fluid
CT: computed tomography
IgE: immunoglobulin E
ITC: itraconazole
VRC: voriconazole
